# SARS-CoV-2 Virion Infectivity and Cytokine Production in Primary Human Airway Epithelial Cells

**DOI:** 10.3390/v14050951

**Published:** 2022-05-02

**Authors:** Thuc Nguyen Dan Do, Sandra Claes, Dominique Schols, Johan Neyts, Dirk Jochmans

**Affiliations:** Department of Microbiology, Immunology and Transplantation, Rega Institute, KU Leuven, 3000 Leuven, Belgium; nguyendanthuc.do@kuleuven.be (T.N.D.D.); sandra.claes@kuleuven.be (S.C.); dominique.schols@kuleuven.be (D.S.)

**Keywords:** SARS-CoV-2, variants of concern, primary human airway epithelial cells, cytokine, organotypic model

## Abstract

The emergence of new SARS-CoV-2 variants and the replacement of preceding isolates have been observed through B.1.1.7, B.1.351, B.1.617.2, and B.1.1.529 lineages (corresponding to alpha, beta, delta, and omicron variants of concern (VoC), respectively). However, there is still a lack of biological evidence to which extent those VoC differ from the ancestral lineages. By exploiting human airway epithelial cell (HAEC) cultures, which closely resemble the human airway architecture and physiology, we report distinctive SARS-CoV-2 tropism in different respiratory tissues. In general, SARS-CoV-2 VoC predominantly infect and replicate in HAEC better than the progenitor USA-WA1 isolate or the BavPat1 isolate, which contains the D614G mutation, even though there is little to no difference between variants regarding their infectivity (i.e., virion-per-vRNA copy ratio). We also observe differential tissue-specific innate immunity activation between the upper and lower respiratory tissues in the presence of the virus. Our study provides better comprehension of the behavior of the different VoC in this physiologically relevant ex vivo model.

## 1. Introduction

Since SARS-CoV-2 was first identified, a new isolate containing the D614G spike (S) substitution was reported with higher infectivity and transmission in in vitro and in vivo models [1]. This 614G virus quickly dominated the global pandemic, especially in Europe. Later, in 2020, the B.1.1.7 (alpha) variant firstly discovered in the UK quickly became dominant worldwide, replacing the 614G lineage before it was usurped by the B.1.162.2 (delta) variant. Meanwhile, B.1.351 (beta) emerged in South Africa and was mainly confined there and its surrounding countries. At the end of 2021, B.1.1.529 (omicron) was first identified in South Africa and rapidly spread to other countries. Epidemiologically, not only is the human-to-human transmission of those isolates more superior than that of other lineages, but their potential escape of vaccine-acquired immunity and adverse impact on therapeutics categorizes them as variants of concern (VoC) [2,3,4,5,6]. Additionally, the delta and alpha variants have been associated with the increase in disease severity [7,8].

New mutations have been detected across VoC’s genomes, both outside and inside the S protein-coding region. As they share amino acid substitutions at the receptor-binding domain (RBD) of the S protein, such as D614G and N501Y, the B.1.1.7 and B.1.351 variants are resistant to the neutralization activity of monoclonal antibodies [9]. Other data indicates that B.1.351 reduces the protective efficacy of current vaccines more considerably than B.1.1.7 [10,11]. The K417N, E848K, and N501Y amino acid changes in key residues of the S protein seem to have greater influence on the neutralization of those variants than other mutations [10]. Overall, VoC and emergent variants [12] with similar mutated residues in the S protein may cause new resurgences of COVID-19 cases worldwide.

To address this, we assessed the susceptibility of different sources of human primary airway epithelial cells (HAEC), cultured at the air–liquid interface (ALI), to authentic alpha, beta, delta, and omicron viruses in comparison with their ancestral lineages. In addition, we analyzed the induction of cytokine production. Our study provides better understanding of the behavior of the different VoC in this physiologically relevant ex vivo model.

## 2. Materials and Methods

### 2.1. SARS-CoV-2 Isolates and Virus Stocks

The ancestral Severe Acute Respiratory Syndrome-related Coronavirus 2 (SARS-CoV-2) strains, Germany/BavPat1/2020 isolate (EPI_ISL_406862|28 January 2020, in this study referred to as BavPat1, kindly provided by Prof. Christian Drosten, University Hospital Charité, Berlin, Germany) and USA/WA-CDC-WA1/2020 isolate (EPI_ISL_404895|19 January 2020, referred to as USA-WA1, provided by BEI Resources) were retrieved from COVID-19 positive patients in early 2020, and have been described previously [13,14]. The USA-WA1 strain is recognized as lineage A and is most similar to the original Wuhan-Hu-1 strain (GeneBank accession number MN908947.3) whereas the BavPat1 is designated as lineage B and carries the D614G amino acid substitution in the spike protein.

The SARS-CoV-2 VoC B.1.1.7 (EPI_ISL_791333|21 December 2020), B.1.351 (EPI_ISL_896474|11 January 2021), B.1.617.2 (EPI_ISL_2425097|20 April 2021), and B.1.1.529 (EPI_ISL_6794907|24 November 2021) isolates were named according to their own lineages. All strains were isolated in-house from nasopharyngeal swabs of travelers returning to Belgium. The generation of virus stock was fully reported elsewhere [15,16,17]. Only early passages were used for the ALI experiment, i.e., passage 3 (P3) of BavPat1, P5 of USA-WA1, P2 of B.1.1.7, B.1.351, B.1.617.2, and B.1.1.529.

All infectious virus-related work was conducted under a biosafety level 3 (BSL-3) facility at the Rega Institute for Medical Research, KU Leuven, according to institutional guidelines.

### 2.2. Cell Culture

The African monkey kidney cell line VeroE6 (ATCC CRL-1586) was cultured in Dulbecco’s modified Eagle’s medium (DMEM; Gibco, catalogue no. 41965-039) supplemented with 10% *v/v* heat-inactivated fetal bovine serum (HI-FBS; HyClone, catalogue no. SV03160.03) and 1% *v/v* Penicillin-Streptomycin 10,000 U/mL (P/S; Gibco, catalogue no. 15140148) at 37 °C and 5% CO_2_. End-point titrations were performed with 2% HI-FBS-containing medium instead of 10%.

### 2.3. Airway Ex Vivo Models and Study Design

A total of 150 air–liquid interface cultures were used in this study. That included 60 human nasal airway epithelial cell cultures (HnAEC; catalogue no. EP02MP) obtained from a pool of fourteen healthy donors (*n* = 9), 30 human bronchial airway epithelial cell cultures (HbAEC; catalogue no. EP01MD) from one donor (*n* = 6), and 60 human small airway epithelial cell cultures (HsAEC; catalogue no. EP21SA) from two different donors (*n* = 12). HAEC were received from Epithelix (Geneva, Switzerland) in an air–liquid interphase set-up. Cultures model the architecture and cellular complexity of the conducting airway at specific regions and are ready-to-use. After arrival, each insert was washed with pre-warmed medium and maintained in corresponding MucilAir medium (Epithelix, catalogue no. EP04MM) or SmallAir medium (Epithelix, catalogue no. EP64SA) at 37 °C and 5% CO_2_ for at least 4 days before use. On the day of the experiment, the HAEC were exposed to 100 µL of SARS-CoV-2 inoculum at the apical side for 1.5 h at 35 °C. The first apical wash with medium was collected 24 h later (day 1 post-infection (p.i.)). Every day from day 0, subsequent apical washes were collected and stored at −80 °C for analysis.

### 2.4. RNA Extraction and Quantitative Reverse Transcription-PCR (RT-qPCR)

Viral RNA in the apical wash was isolated using the Cells-to-cDNA^TM^ II cell lysis buffer kit (Thermo Fisher Scientific, Merelbeke, Belgium, catalogue no. AM8723). Briefly, 5 µL wash fluid was added to 50 µL lysis buffer, incubated at room temperature (RT) for 10 min and then at 75 °C for 15 min. 150 µL nuclease-free water was additionally added to the mixture prior to RT-qPCR. In parallel, a ten-fold serial dilution of 2019_nCoV_N_positive control from IDT Technologies (catalogue no. 10006625) was used to generate a standard curve. The amount of viral RNA expressed as number of copies per mL (copies/mL) was quantified by RT-qPCR on a LighCycler96 platform (Roche, Anderlecht, Belgium) using the iTaq universal probes one-step kit (Bio-Rad, Temse, Belgium, catalogue no. 1725141) and a commercial mix of primers for the N gene (forward primer 5′-GACCCCAAAATCAGCGAAAT-3′, reverse primer 5′-TCTGGTTACTGCCAGTTGAATCTG-3′) and probes (5′-FAM-ACCCCGCATTACGTTTGGTGGACC-BHQ1-3′) manufactured at IDT Technologies (catalogue no. 10006606). The reaction was prepared according to the manufacturer’s protocol.

### 2.5. End-Point Titration Assay

VeroE6 cells were seeded in 96-well tissue culture plates (Corning, Amsterdam, The Netherlands, catalogue no. 353072) at a density of 1 × 10^4^ cells per well. After 24 h, serial dilutions of ALI wash fluid were prepared in the plates. Cells were incubated for 3 days at 37 °C and evaluated microscopically for the absence or presence of the virus-induced cytopathic effect (CPE). The infectious viral titer was determined by end-point titration, expressed as 50% tissue culture infectious dose per mL (TCID_50_/mL). Virus titers were calculated by using the Reed and Muench method as previously reported [18].

### 2.6. Cell Viability Assay

At the end of the experiment, HAEC were washed once with pre-warmed 1× PBS (Thermo Fisher Scientific, catalogue no. 14190-094) before being incubated with 200 µL 0.25% trypsin-EDTA (Thermo Fisher Scientific, catalogue no. 25200056) at 37 °C for 2 h. Single cell suspension was stained with acridine orange and propidium iodide (AOPI) solution. The number of living cells and dead cells were automatically counted by a Luna^TM^ automated cell counter (Logos Biosystems, Villeneuve d’Ascq, France)), giving the cell viability of HAEC as the percentage of living cells over total cell numbers.

In case of nasal HAEC cell growth was determined via a colorimetric method using the CYQUANT^TM^ LDH Cytotoxicity Assay Kit (Thermo Fisher Scientific, catalogue no. C20301). After being washed apically, cultures were refreshed with new basolateral medium and lysed for 2 h. The activity of released lactate dehydrogenase (LDH) in the basal medium was proportionally quantified through the level of formazan formation at 490 nm. Cell viability was expressed as the percentage of LDH activity in infected groups compared to the non-infected one.

### 2.7. Profiling of Chemokine and Cytokine Secretion

Apical wash fluid and/or basolateral medium were analyzed for a panel of chemokines and cytokines—IP-10, IL-6, IL-10, IFN-ɣ, and TNF-α using customized ProcartaPlex assay kits (Thermo Fisher ScientificInvitrogen) according to the manufacturer’s protocol. Those cytokines have been reported as being linked to disease exacerbation in patients with COVID-19 [19]. The analyte concentrations were determined using the included standard curve of known concentrations coupled with the Luminex XPONENT v4.2 software.

### 2.8. Statistical Analysis

The number of cultures and independent experiments that were performed is indicated in each legend of the individual figure. Results are represented as median and 95% confidence intervals for cytokine and chemokine expressions or mean ± standard deviation in other experiments. All statistical comparisons in the study were performed in GraphPad Prism v9.2.0 (GraphPad Software, San Diego, CA, USA). Statistical significance was determined using Kruskal–Wallis with Dunnett’s multiple comparison test unless specifically mentioned otherwise. *p*-values of ≤0.05 were considered statistically significant. In the figures, “ns” indicates a *p* > 0.05 and an asterisks indicates a statistical significance level of * *p* < 0.05, ** *p* < 0.01, and *** *p* < 0.001.

## 3. Results

**Figure 1:**
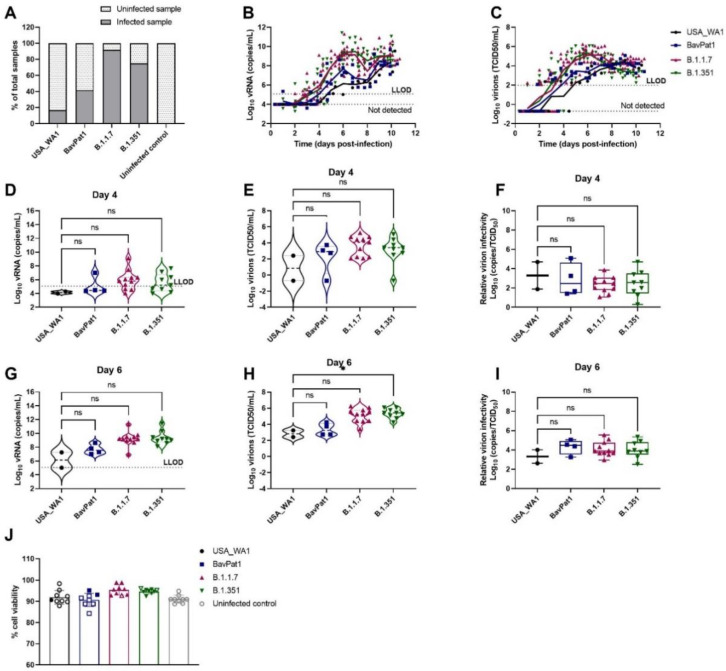
SARS-CoV-2 VoC replication kinetics in human small airway epithelial cell cultures. Viral replication of SARS-CoV-2 VoC at the viral input of 2 × 10^3^ TCID_50_/insert in HsAEC cultures. The cultures were incubated at 35 °C and maintained for 10 days. (**A**) Percentages of infected cultures in total tested samples. Two independent experiments with HsAEC from two different donors were performed. Six independent inserts were used for each condition per experiment. (**B**,**D**,**G**) Viral RNA or (**C**,**E**,**H**) infectious particles in apical washes of infected cultures were determined by RT-qPCR or by end-point titration assay, respectively. (**F**,**I**) Relative virion infectivity (i.e., the ratio of the number infectious particles over the number of viral RNA). (**J**) Cell viability of HsAEC cultures. Infected samples were represented as closed markers whereas non-infected samples were indicated as open markerss. Dots represent individual samples. The dotted lines indicate the lower limit of detection (LLOD). ns *p* > 0.05, * *p* < 0.05.

To investigate the relative infectivity and cytokine secretion profile of VoC B.1.1.7 and B.1.351, we infected HAEC from a small airway origin (HsAEC) with 2 × 10^3^ TCID50 of each SARS-CoV-2 isolate including the ancestral isolates USA-WA1 and BavPat1. As expected, only 17% of the cultures (2 out of 12) inoculated with the USA-WA1 isolate resulted in productive infection while 42%, 92%, and 75% of samples showed a productive infection when inoculated with BavPat1, B.1.1.7, and B.1.351 isolates, respectively (*n* = 12) (Figure 1A). Only infected samples were presented in the next figures. B.1.1.7 and B.1.351 variants replicated more efficiently during the first 6 days of the experiment, reaching a peak of 10^9^ RNA copies/mL (Figure 1B) and 10^5^ TCID_50_/mL (Figure 1C) while only 10^6^ and 10^7^ RNA copies/mL and ~10^3^ TCID_50_/mL were recorded for the comparators (USA-WA1 and BavPat1, respectively). Although it was noticed that infectious particles were significantly produced on day 6 in B.1.351-infected inserts (*p* = 0.0476; Figure 1H), no difference in relative infectivity was observed for all four VoC (*p* = 0.9181; Figure 1F) (*p* = 0.7611; Figure 1I). All HAEC-cultures maintained more than 90% cell viability (Figure 1J).

**Figure 2:**
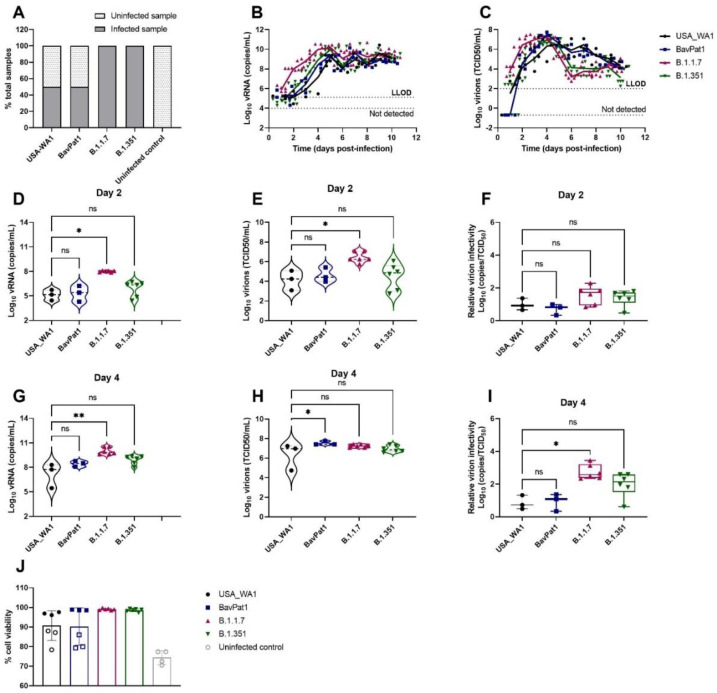
SARS-CoV-2 VoC replication kinetics in human bronchial airway epithelial cell cultures. Viral replication of SARS-CoV-2 VoC at the viral input of 2 × 10^3^ TCID_50_/insert in HbAEC cultures. The cultures were incubated at 35 °C and maintained for 10 days. (**A**) Percentages of infected cultures in total tested samples. Six independent inserts were used for each infected condition while only four inserts were used for non-infected control group. (**B**,**D**,**G**) Viral RNA or (**C**,**E**,**H**) infectious particles in apical washes of infected cultures were determined by RT-qPCR or by end-point titrations, respectively. (**F**,**I**) Relative virion infectivity. (**J**) Cell viability of HbAEC cultures. Infected samples were represented as filled markers whereas non-infected samples were indicated as open markers. Dots represent individual samples. The dotted lines indicate the lower limit of detection (LLOD). ns *p* > 0.05, * *p* < 0.05, ** *p* < 0.01.

Likewise, a similar observation was made using HAEC of bronchial origin (HbAEC) (Figure 2). Whereas six out of six samples were infected by either the B.1.1.7 or the B.1.351 variant, only 50% of the cultures could be infected with either USA-WA-1 or BavPat1 isolates (*n* = 6) (Figure 2A). At 2 d.p.i., the viral RNA loads were determined at 2 × 10^5^, 7 × 10^5^, 1 × 10^8^, and 2 × 10^6^ RNA copies/mL (Figure 2D) and the infectious virus titers were 5 × 10^4^, 1 × 10^5^, 5 × 10^6^, and 3 × 10^5^ TCID_50_/mL (Figure 2E) in USA-WA1, BavPat1, B.1.1.7, and B.1.351 groups, respectively. As a result, no disparity in the virion infectivity was observed (*p* = 0.1169; Figure 2F). Of note, the alpha-infected cultures produced a lower infectivity ratio in comparison to the ancestral strain USA-WA1 on day 4 p.i. (*p* = 0.0162; Figure 2I). Cultures incubated with any isolate maintained at least 90% cell viability after the 10-day experiment (Figure 2J).

**Figure 3:**
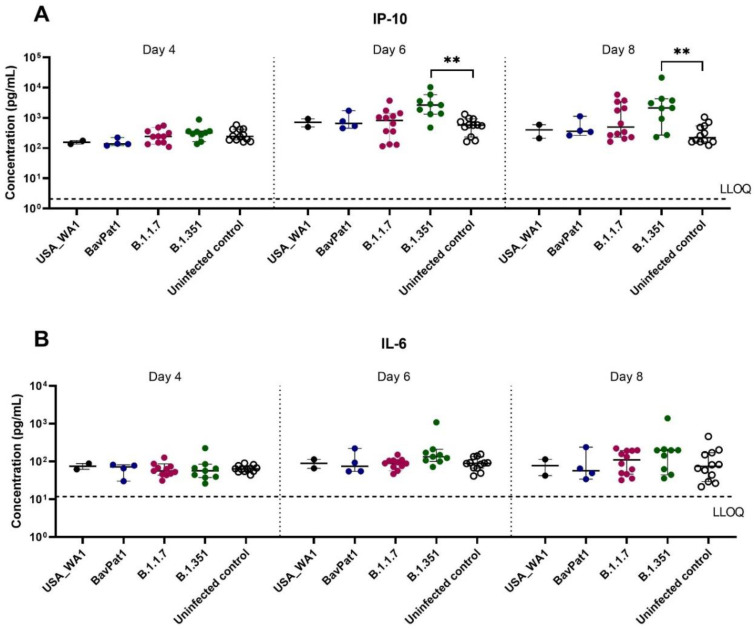
Cytokine and chemokine expression profiles after infection of HsAEC with different SARS-CoV-2 isolates. Production of (**A**) IP-10 or (**B**) IL-6 in HsAEC post-infection in the presence or absence of SARS-CoV-2. For this analysis, the infected samples from two experiment samples were pooled. The dotted lines represent the lower limit of quantification (LLOQ). Statistical significance between variants and uninfected control was calculated by Kruskal–Wallis with two-sided Dunn’s post hoc test. ** *p* < 0.01.

**Figure 4:**
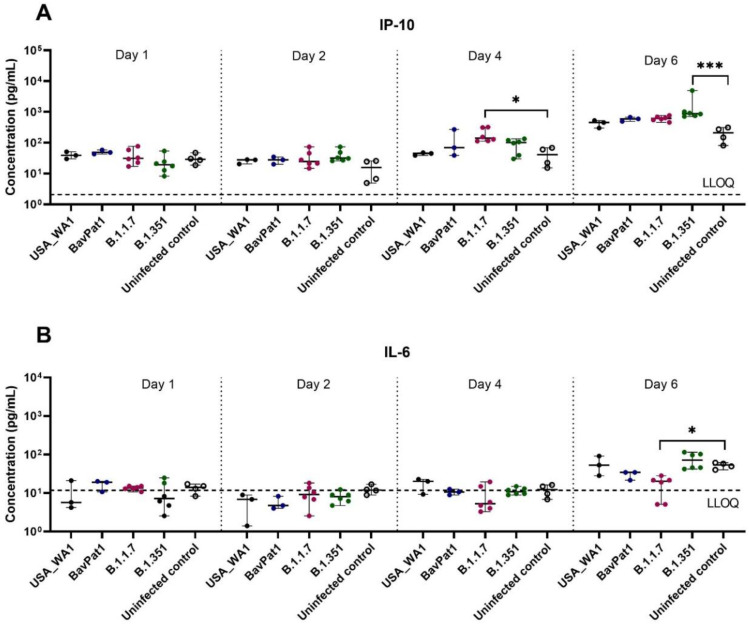
Cytokine and chemokine expression profiles after infection of HbAEC with different SARS-CoV-2 isolates. Production of (**A**) IP-10 or (**B**) IL-6 in HbAEC post-infection in the presence or absence of SARS-CoV-2. The dotted lines represent the lower limit of quantification (LLOQ). Statistical significance between the variants and uninfected control was calculated by Kruskal–Wallis with two-sided Dunn’s post hoc test. * *p* < 0.05, *** *p* < 0.001.

To further comprehend the immune response of the HAEC towards SARS-CoV-2 infection, the expression levels of cytokines related to COVID-19 in patients were analyzed in the apical washes of the cultures. Only for the B.1.351 and B.1.1.7 isolates we could see a difference in cytokine production compared to the uninfected controls. In the B.1.351-infected group, an upregulation of IP-10 levels of 5.5 fold (*p* = 0.0044; Figure 3A) to 7.5 fold (*p* = 0.0004; Figure 4A) compared to the uninfected group was observed at 6 days p.i. A 4.6-fold increase in IP-10 expression was induced by the B.1.1.7 variant on day 4 p.i. (*p* = 0.0114; Figure 4A). No upregulation in IL-6 production was observed (Figure 3B, Figure 4B). Likewise, no difference in other cytokine levels (i.e., IL-10, IFN-ɣ, TNF-α) was noted (data not shown). The overall discrepancy in cytokine production between the alpha and beta variants versus basal strains was limited.

**Figure 5:**
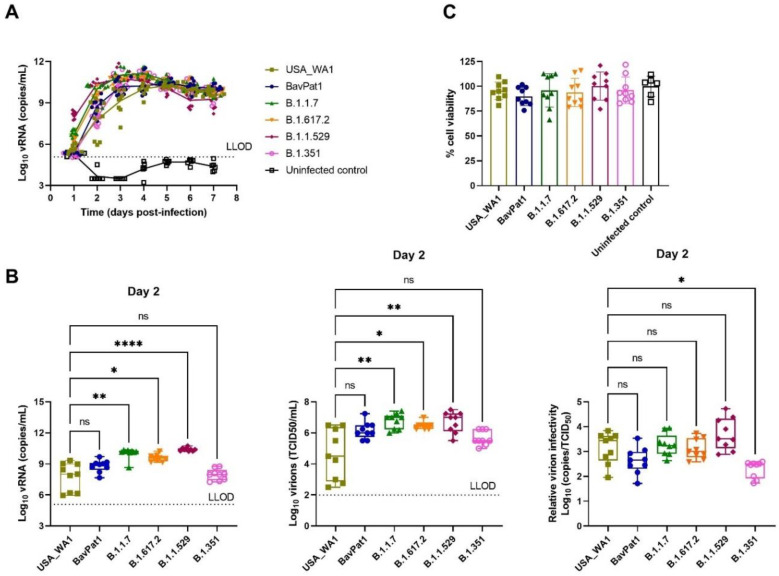
The replication profile of different SARS-CoV-2 isolates in nasal HAEC. The nasal tissues were infected with SARS-CoV-2 at 2 × 10^3^ TCID_50_/culture at 35 °C for 7 days. (**A**) Viral loads from the apical washes are expressed as log_10_ SARS-CoV-2 RNA copies per mL. (**B**) Viral RNA copies, infectious particles, and relative virion infectivity at 2 d.p.i. are expressed as log_10_ RNA copies per mL, log_10_ TCID50 per mL, and log_10_ inverse ratio of TCID50-per-copy, respectively. (**C**) Cell viability of HnAEC after 7 days p.i. Each dot represents individual data. The lower limit of detection (LLOD) is indicated as dotted lines. Statistical significance was determined using Kruskal–Wallis with Dunnett’s multiple comparison test with ns *p* > 0.05, * *p* < 0.05, ** *p* < 0.01, **** *p* < 0.0001.

**Figure 6:**
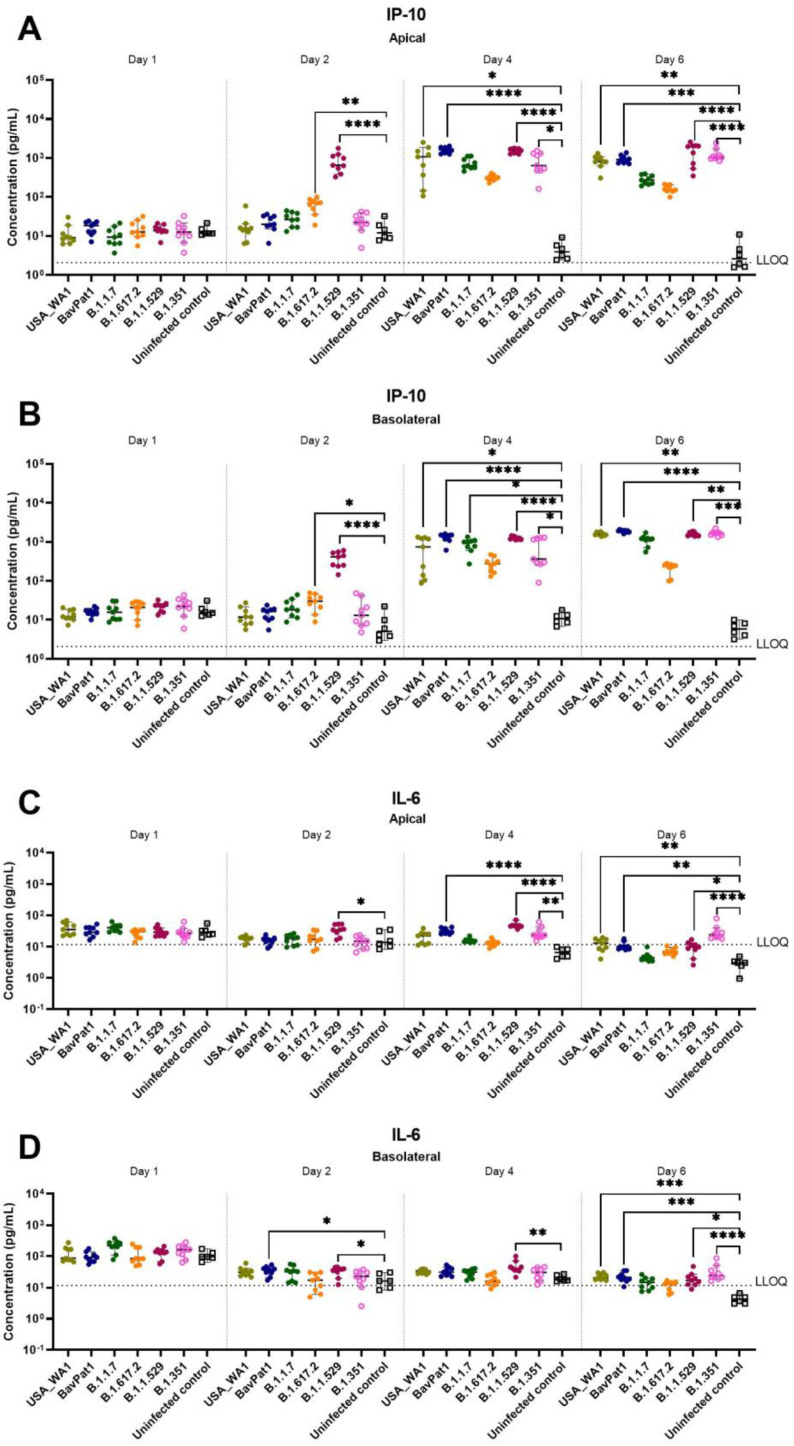
The expression of COVID-19-related chemokine and cytokine in HnAEC. Concentrations of IP-10 (**A**,**B**) and IL-6 (**C**,**D**) in the presence or absence of SARS-CoV-2 are expressed as pg per mL. Mean values with 95% CI are shown and statistical significance between the variants and non-infected control was calculated by Kruskal–Wallis with two-sided Dunn’s post hoc test. * *p* < 0.05, ** *p* < 0.01, *** *p* < 0.001, **** *p* < 0.0001.

Since the delta (B.1.617.2) and omicron (B.1.1.529) variants became dominant worldwide in mid and late 2021, replacing the preceding isolates in most countries, we performed an additional experiment exploring SARS-CoV-2 VoC infection using HAEC from nasal regions. After 24-h p.i. (h.p.i.), viral RNA of all SARS-CoV-2-infected groups were detectable with a large replication advantage in omicron-infected samples (Figure 5A). At 2 d.p.i., the omicron isolate produced the highest quantity of vRNA (2.5 × 10^10^ RNA copies/mL, *p* < 0.0001) and virions (5 × 10^6^ TCID_50_/mL, *p* = 0.0066) when compared with USA-WA1 (6.3 × 10^7^ RNA copies/mL and 4 × 10^4^ TCID_50_/mL) and the alpha (10^10^ RNA copies/mL, *p* = 0.0020; 6.3 × 10^6^ TCID_50_/mL, *p* = 0.0041) and delta (4 × 10^9^ RNA copies/mL, *p* = 0.0366; 3 × 10^6^ TCID_50_/mL, *p* = 0.0303) isolates (Figure 5B). Again, comparable infectivity of the virions produced by the different groups was noted (Figure 5B). There was no influence of SARS-CoV-2 infection on HnAEC cell viability (Figure 5C). Interestingly, infection with either omicron, delta, or BavPat1 led to an upregulation of IP-10 and/or IL-6 expression in apical washes and/or basal medium compared to non-infected samples at 48 h.p.i. (Figure 6). For instance, IP-10 and IL-6 levels were both induced in the B.1.1.529-infected group while only the former was upregulated in B.1.617.2-infected cultures, with a statistical significance compared to the negative control (*p* < 0.0001 for B.1.1.529/IP-10, *p* = 0.0179–0.0068 for B.1.617.2/IP-10, *p* = 0.0429–0.0469 for B.1.1.529-IL-6). From day 4 p.i., the production of IP-10 and IL-6 were markedly accelerated in most of SARS-CoV-2-infected cultures, but not in the alpha and delta ones. Taken together, comparable infectivity was observed between samples infected with VoC and ancestral isolates, and alpha, beta, omicron, and delta variants have different effects on cytokine secretions in the human respiratory tract (HRT) at the onset of SARS-CoV-2 infection.

By comparing the replication kinetics of SARS-CoV-2 strains in different inserts generated from distinctive regions of the HRT, we observed that relative virion infectivity was not only tissue-dependent (*p* < 0.0001; two-way ANOVA) but also SARS-CoV-2 variant-dependent (*p* = 0.0030, two-way ANOVA). As infected samples from small airway tissues did not effectively produce detectable RNA copies and/or infectious progenies after 2 d.p.i., we excluded this data from the HsAEC experiments for further analysis.

**Figure 7:**
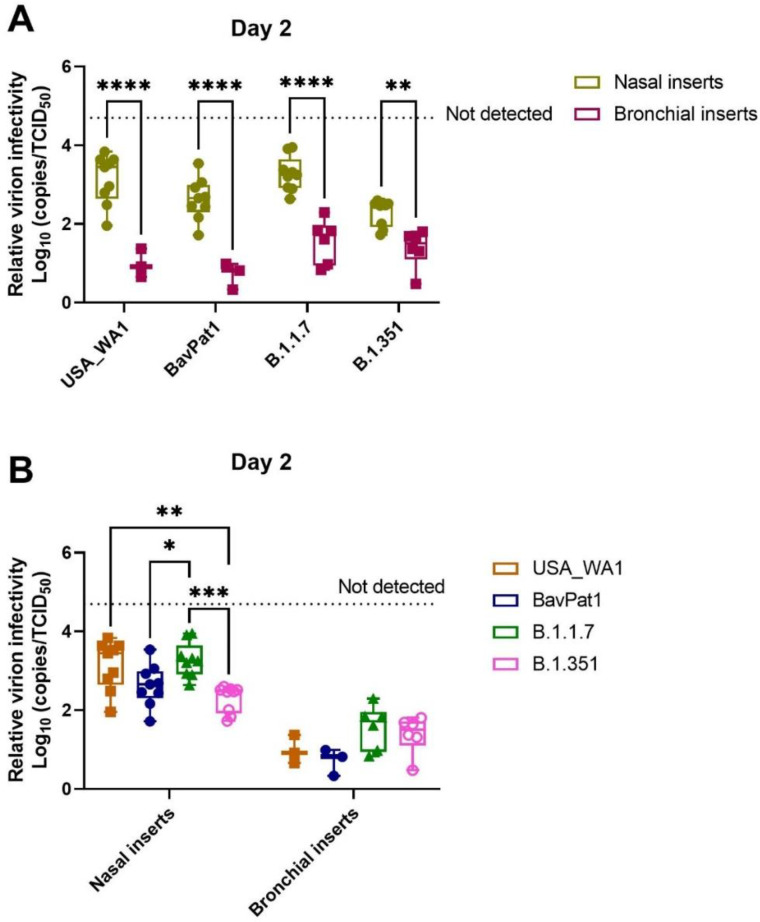
Comparison of SARS-CoV-2 virion infectivity in different human respiratory tissues. For this analysis, infected samples on day 2 p.i. from independent experiments were pooled. The relative infectivity is expressed as log_10_ inverse ratio of TCID_50_-per-copy. A comparison between the different cell types (**A**) as well as a comparison between the different VoCs (**B**) is shown. Each dot represents individual cultures and statistical significance was determined by two-way ANOVA with a Turkey post-hoc test. * *p* < 0.05, ** *p* < 0.01, *** *p* < 0.001, **** *p* < 0.0001.

In general, bronchial tissues produced a higher virion-per-vRNA copy ratio (around 1–2 logs increase) than the nasal tissues (Figure 7A, Table 1). In addition, the beta variant generated higher infectivity than the alpha or USA-WA1 whilst the omicron isolate produced the lowest ratio of virion-per-vRNA copy in nasal cultures. The VoC (alpha and beta) were inclined to have lower virion:vRNA copy levels than the ancestral lineages, but no difference between isolates was noted in bronchial samples (Figure 7B).

## 4. Discussion

The human respiratory tract (HRT), the primary target of SARS-CoV-2 infection, extends from the nasal cavity to the terminal bronchioles and is fully covered by the airway epithelium. By forming a pseudostratified epithelial layer, the organotypic HAEC-model closely resembles the human airway architecture and physiology with its dynamic cellular composition including basal cells, ciliated cells, and goblet cells [20]. Although SARS-CoV-2 genetic diversity rapidly evolved, little is known about the comparison of VoC infection and virulence in a physiologically relevant human model, potentially explaining the current epidemiological observations. Here, we report distinctive SARS-CoV-2 tropism in different respiratory tissues. In general, the SARS-CoV-2 VoC predominantly infects and replicates in HAEC better than the ancestral USA-WA1 isolate or the ancestral BavPat1 isolate, which contains the D614G mutation. A possible explanation is that the heavily mutated spike (S) protein, including the P681R amino acid change in the delta variant, enhances the cleavage efficiency of S1 and S2 subunits, resulting in more productive virus entry into airway epithelial cells, thereby boosting the replication fitness of the mutant [21]. Another S substitution (i.e., N501Y seen in alpha and beta isolates) also confers a major fitness advantage in the upper airway replication and transmission via an increased receptor-binding domain (RBD)/angiotensin-converting enzyme 2 (ACE2) interaction [22]. Mutations in the nucleocapsid protein (R203K and G204R observed in omicron) is associated with increased viral replication [23]. To this end, our finding is in line with other reports [21,22,24,25]. In particular, the extremely fast replication of omicron could be due to its adapted cellular tropism (less transmembrane protease serine 2 (TMPRSS2) dependent), allowing the virus to infect more nasal cells via the endocytic cathepsin L pathway than the competitors [24,25,26].

When it comes to their infectivity (i.e., virion-per-vRNA copy ratio), there is little to no difference between variants in comparison with the ancestral USA-WA1 lineage. One possible reason for the discrepancy concerning the virion-per-vRNA copy ratio observed in alpha-infected cultures (Figure 2I) is that other undefined mutations around the furin cleavage site (e.g., T716I, N679K) modulate the proteolytic cleavage of the alpha S protein, thus, destabilizing the S trimer conformation, leading to unstable virions [26]. Our observation is in accordance with the replicative fitness of alpha lineage in HAEC previously reported [27]. Of note, since not all extracellular vRNA represents equal infectious progeny, it is of importance to compare the viral infectivity based on the virion:vRNA copy value rather than the sole parameter of vRNA copy or infectious titer. This perhaps gives better insights into the transmission dynamics of viral pathogens [28]. The lower virion:vRNA copy ratio is often attributed to incomplete genomes, lethal mutations, deficits in virion capsid/envelope structure, or host barriers which restrict early viral replication [29]. As the new SARS-CoV-2 variants evolve to more productively enter the airway epithelial cells [21,22], it is unclear why their virion-per-copy ratios are comparable to that of the progenitor USA-WA1 and to what extent that might explain the corresponding human-to-human transmission. Therefore, further experiments are required to elucidate the stability of VoC virion structure and its corresponding infectivity to explain the similar virion:vRNA copy observed in this study.

The epithelial composition of different anatomical regions in HRT has also been reported to partly influence SARS-CoV-2 replication and spread [20]. High co-expression levels of *ACE2* and *TMPRSS2* (two main entry receptors for SARS-CoV-2 infection) have been found in both secretory and ciliated cells [30,31], facilitating SARS-CoV-2 replication [32]. Notably, the co-expression of *ACE2* and *TMPRSS2* genes were more enriched in the nasal cavity than in the lower airway and parenchyma [31]. This explains why the highest percentage (100%) of infected samples in our study is from nasal tissue, followed by bronchial and small airway tissues.

In the current study, we observed the upregulation of proinflammatory cytokines and chemokines (IL-6, IP-10) in omicron-inoculated nasal tissues 48 h.p.i. This could be due to the extremely high viral replication of omicron already on day 1 p.i. Later, elevated production of IP-10 and IL-6 was also observed in other groups at a similar level, suggesting a saturated condition. A lesser influence on protein secretions was noted in the alpha and delta groups, implying variant-specific immune responses. Meanwhile, the high concentrations of IL-6 and IP-10 in patients’ sera, together with the infiltrating macrophages, have been depicted as the “immune signature” of SARS-CoV-2 and as the prototype factors driving COVID-19 progression [33]. On the other hand, it is reported that the systemic and mucosal immunity show distinct responses to SARS-CoV-2 infection, with a strong impact on the nasalpharyngeal cytokine profile [34]. This could perhaps explain the unusual cytokine release profile in nasal tissues, which is probably not the source of cytokines in the plasma of COVID-19 patients. In small airway and bronchial cultures, the induction of the epithelium-secreting IP-10 protein is not in accordance with the secretion of the IL-6 protein. Taken together, it is plausible that epithelial cells are conditioned to produce immune-associated proteins to reduce viral susceptibility. The restricted innate immune response to SARS-CoV-2 reported herein is in agreement with data from another study [35] and expands the observations in cytokine secretion in primary HAEC and in lung explants [35,36,37]. In this regard, the differential tissue-specific innate immune activation between the upper and lower respiratory tissues could also be attributed to the discrepancy in observed virion infectivity.

One of the limitations of our study is the lack of a direct competition experiment, which could expose replicative differences not detected in single growth kinetic assays, as previously reported [21,22,25,27,38].

## 5. Conclusions

To conclude, our findings reveal that despite the comparable virion infectivity, the different SARS-CoV-2 VoC are endowed with faster and enhanced replication competence in HAEC cultures compared with ancestral lineages, explaining their more efficient transmission in the human population. Moreover, a distinctive tropism of respiratory tissues in response to SARS-CoV-2 infection is a pivotal determinant in elucidating host–pathogen interactions or selecting a suitable model for antiviral testing.

## Figures and Tables

**Table 1:** Descriptive summary of log_10_ inverse ratio of TCID_50_-per-vRNA copy.

Insert Origin	SARS-CoV-2 Strain	Mean	Standard Deviation	*N*
Nasal inserts	USA-WA1	3.14	0.63	9
	BavPat1	2.63	0.53	9
	B.1.1.7	3.27	0.44	9
	B.1.351	2.30	0.34	9
	B.1.617.2	3.10	0.41	9
	B.1.1.529	3.71	0.64	9
Bronchial inserts	USA-WA1	0.98	0.36	3
	BavPat1	0.71	0.34	3
	B.1.1.7	1.56	0.56	6
	B.1.351	1.38	0.48	6

## Data Availability

Data are contained within the article.
